# Masitinib is a broad coronavirus 3CL inhibitor that blocks replication of SARS-CoV-2

**DOI:** 10.1126/science.abg5827

**Published:** 2021-08-20

**Authors:** Nir Drayman, Jennifer K. DeMarco, Krysten A. Jones, Saara-Anne Azizi, Heather M. Froggatt, Kemin Tan, Natalia Ivanovna Maltseva, Siquan Chen, Vlad Nicolaescu, Steve Dvorkin, Kevin Furlong, Rahul S. Kathayat, Mason R. Firpo, Vincent Mastrodomenico, Emily A. Bruce, Madaline M. Schmidt, Robert Jedrzejczak, Miguel Á. Muñoz-Alía, Brooke Schuster, Vishnu Nair, Kyu-yeon Han, Amornrat O’Brien, Anastasia Tomatsidou, Bjoern Meyer, Marco Vignuzzi, Dominique Missiakas, Jason W. Botten, Christopher B. Brooke, Hyun Lee, Susan C. Baker, Bryan C. Mounce, Nicholas S. Heaton, William E. Severson, Kenneth E. Palmer, Bryan C. Dickinson, Andrzej Joachimiak, Glenn Randall, Savaş Tay

**Affiliations:** 1 Pritzker School for Molecular Engineering, The University of Chicago, Chicago, IL, USA.; 2 Center for Predictive Medicine for Biodefense and Emerging Infectious Diseases, University of Louisville, Louisville, KY, USA.; 3 Department of Chemistry, The University of Chicago, Chicago, IL, USA.; 4 Department of Molecular Genetics and Microbiology, Duke University, Durham, NC, USA.; 5 Center for Structural Genomics of Infectious Diseases, Consortium for Advanced Science and Engineering, University of Chicago, Chicago, IL, USA.; 6 Structural Biology Center, X-ray Science Division, Argonne National Laboratory, Argonne, IL, USA.; 7 Cellular Screening Center, The University of Chicago, Chicago, IL, USA.; 8 Department of Microbiology, Ricketts Laboratory, University of Chicago, Chicago, IL, USA.; 9 Department of Microbiology and Immunology, Stritch School of Medicine, Loyola University Chicago, Maywood, IL, USA.; 10 Department of Medicine, Division of Immunobiology, Larner College of Medicine, University of Vermont, Burlington, VT, USA.; 11 Department of Microbiology and Molecular Genetics, Larner College of Medicine, University of Vermont, Burlington, VT, USA.; 12 Department of Molecular Medicine, Mayo Clinic, Rochester, MN, USA.; 13 Department of Ophthalmology and Visual Sciences, Illinois Eye and Ear Infirmary, College of Medicine, University of Illinois at Chicago, Chicago, IL, USA.; 14 Institut Pasteur, Viral Populations and Pathogenesis Unit, Centre National de la Recherche Scientifique UMR 3569, Paris, France.; 15 Vaccine Testing Center, Larner College of Medicine, University of Vermont, Burlington, VT, USA.; 16 Department of Microbiology, University of Illinois at Urbana-Champaign, Urbana, IL, USA.; 17 Carl R. Woese Institute for Genomic Biology, University of Illinois at Urbana-Champaign, Urbana, IL, USA.; 18 Department of Pharmaceutical Sciences, College of Pharmacy, Biophysics Core at Research Resources Center, University of Illinois at Chicago, Chicago, IL, USA.; 19 Department of Biochemistry and Molecular Biology, The University of Chicago, Chicago, IL, USA.

## Abstract

There is an urgent need for antiviral agents that treat severe acute respiratory syndrome coronavirus 2 (SARS-CoV-2) infection. We screened a library of 1900 clinically safe drugs against OC43, a human beta coronavirus that causes the common cold, and evaluated the top hits against SARS-CoV-2. Twenty drugs significantly inhibited replication of both viruses in cultured human cells. Eight of these drugs inhibited the activity of the SARS-CoV-2 main protease, 3CLpro, with the most potent being masitinib, an orally bioavailable tyrosine kinase inhibitor. X-ray crystallography and biochemistry show that masitinib acts as a competitive inhibitor of 3CLpro. Mice infected with SARS-CoV-2 and then treated with masitinib showed >200-fold reduction in viral titers in the lungs and nose, as well as reduced lung inflammation. Masitinib was also effective in vitro against all tested variants of concern (B.1.1.7, B.1.351, and P.1).

## Targeting the main protease of SARS-CoV-2

Inside host cells, the RNA genome of severe acute respiratory syndrome coronavirus 2 (SARS-CoV-2) is translated into two polyproteins that are cleaved to give the individual viral proteins. The main viral protease, known as Mpro or 3CLpro, plays a key role in these cleavages, making it an important drug target. Drayman *et al*. identified eight drugs that target 3CLpro from a library of 1900 clinically safe drugs. Because of the challenge of working with SARS-CoV-2, they started by screening for drugs that inhibit the replication of a human coronavirus that causes the common cold. They then evaluated the top hits for inhibiting SARS-CoV-2 replication and for inhibiting 3CLpro. Masitinib, a broad antiviral, inhibited the main proteases of coronaviruses and picornaviruses and was effective in reducing SARS-CoV-2 replication in mice. —VV

## Teaser

A repurposing screen shows that the orally available drug masitinib is able to inhibit virus replication in mouse-based models of COVID-19.

In January 2020, severe acute respiratory syndrome coronavirus 2 (SARS-CoV-2) was identified as the causative agent of a new respiratory syndrome that was later named COVID-19 (*1*). The virus has rapidly spread throughout the world, causing an ongoing pandemic, with millions of deaths (*2*). SARS-CoV-2 is a member of *Coronaviridae*, a family of enveloped, single-strand, positive-sense RNA viruses (*3*). This family is composed of both human and animal pathogens, including two other emerging human pathogens [SARS-CoV and Middle East respiratory syndrome coronavirus (MERS-CoV)] as well as four endemic human viruses that are the second most common cause of the common cold (HCoV-OC43, 229E, NL63, and HKU1) (*4*).

Upon entry into the host cell cytoplasm, the viral genome is translated into roughly 30 proteins. Of these, 16 are initially translated as two polyproteins that must be cleaved into the individual viral proteins for infection to proceed. This cleavage is mediated by two virally encoded proteases: the main viral protease, known as Mpro, 3CLpro, or nonstructural protein 5 (nsp5); and a second protease known as the papain-like protease, PLpro, a domain within nsp3 (*3*). There is interest in developing de novo inhibitors to target these proteases (*5*–*10*), but this is a lengthy process.

Although several vaccines received emergency use authorization from health authorities worldwide and are being deployed, it will take a long time to vaccinate the world population, and the emergence of viral escape mutants that render vaccines ineffective remains a possibility. Therefore, there is a continued need for new treatment options for COVID-19, as well as for broad-spectrum antivirals that could be used against future emerging viruses. Remdesivir, an RNA-dependent RNA-polymerase inhibitor, has been reported to shorten COVID-19 hospitalization times (*11*), but it failed a large clinical trial in hospitalized patients (*12*) and its efficacy is unclear.

Drug-repurposing screens have been used to identify safe-in-human drugs with potential anti–SARS-CoV-2 properties (*9*, *13*, *14*). Repurposed drugs that have existing clinical data on the effective dose, treatment duration, side effects, and toxicity could be rapidly translated into the treatment of patients.

**Fig. 1.**
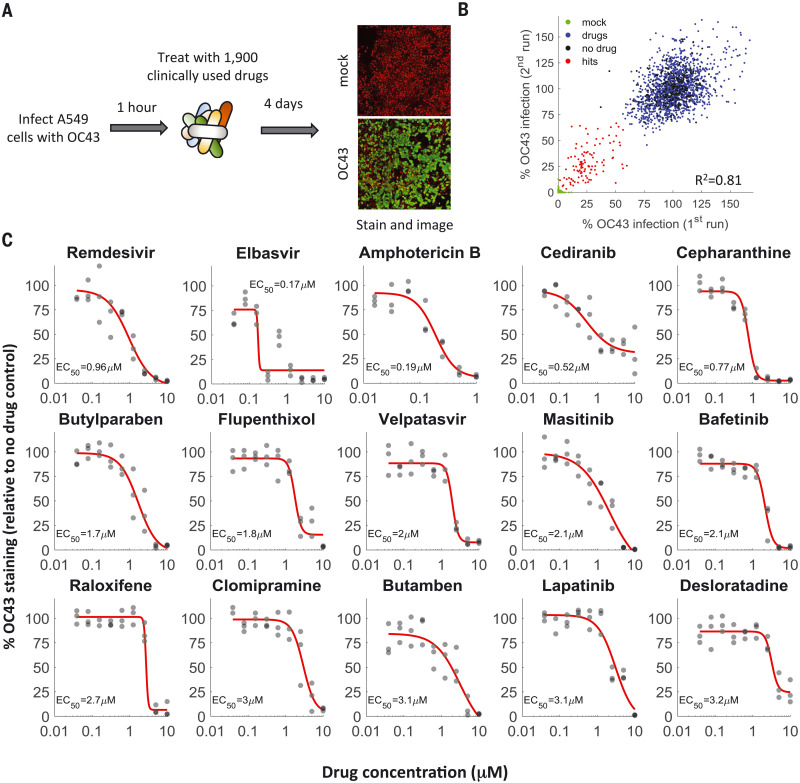
A drug-repurposing screen identifies multiple safe-in-human drugs that inhibit OC43 infection. (**A**) Schematic of the screen. A549 cells expressing H2B-mRuby were infected with OC43 (MOI 0.3), treated with drugs, incubated for 4 days at 33°C, and stained for the viral nucleoprotein. (**B**) Screen results showing the percentage of OC43 staining of mock-infected cells (green), no-drug controls (black), drugs with no effect on OC43 infection (blue), and screen hits (red). Overall agreement between the two repeats is high [coefficient of determination (*R*^2^) = 0.81]. (**C**) Dose-response curves of remdesivir and the top hits from the screen; *n* = 3. Individual measurements are shown as semitransparent gray circles. (Note that some circles overlap.) Additional dose-response curves are shown in fig. S1.

We screened a library of 1900 clinically used drugs, either approved for human use or with extensive safety data in humans (phase 2 or 3 clinical trials), for their ability to inhibit infection of A549 cells by OC43. We chose OC43 because it is a human pathogen that belongs to the same clade of beta coronaviruses as SARS-CoV-2 and can be studied under “regular” biosafety conditions, as well as in an attempt to discover broad-spectrum anti-coronavirus drugs that would be beneficial against SARS-CoV-2 and future emerging coronaviruses. One day after plating, cells were infected at a multiplicity of infection (MOI) of 0.3 and incubated at 33°C for 1 hour, and drugs were added to a final concentration of 10 μM. Cells were then incubated at 33°C for 4 days, fixed, and stained for the presence of the viral nucleoprotein (Fig. 1A). We imaged the cells at day zero (after drug addition) and day four (after staining) to determine the effect of the drugs on cell growth and OC43 infection.

We repeated the screen twice and identified 108 drugs that significantly reduced OC43 infection (Fig. 1B and table S1). For further validation, we looked at the top 35 hits, chose one drug in cases where it was tested in different formulations (such as erythromycin cyclocarbonate and erythromycin estolate), and excluded drugs that were already evaluated against COVID-19 and found ineffective (such as chloroquine) or that were withdrawn due to toxicity (such as mesoridazine). We additionally included trimipramine, which was not present in our screen, because two closely related drugs (imipramine and clomipramine) were top hits. We determined the median effective concentration (EC_50_) values (drug concentration required to reduce infection by 50%) of these 29 drugs against OC43 infection (Fig. 1C and fig. S1) as well as their effect on cell proliferation (CC_50_; fig. S2). With the exception of erythromycin, all drugs inhibited OC43 infection in a dose-dependent manner, with EC_50_ values ranging from 0.17 to 7 μM.

**Fig. 2.**
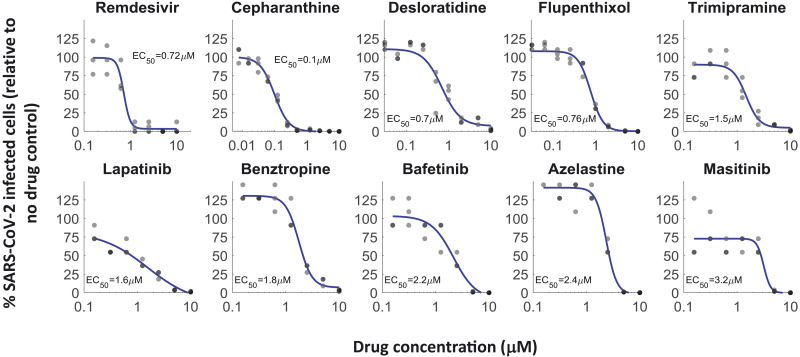
Discovery of repurposed drugs that inhibit SARS-CoV-2 in human lung cells. Of the 26 drugs that inhibited OC43 and tested against SARS-CoV-2, 20 inhibited SARS-CoV-2 replication in a dose-dependent manner, showing good concordance between OC43 and SARS-CoV-2 inhibition. A549 cells overexpressing ACE2 were pretreated with the indicated drugs for 2 hours, infected with SARS-CoV-2 (MOI 0.5), and incubated for 2 days. Cells were stained for the presence of the spike protein, and the percentage of infected cells was determined. Most of the drugs that were effective against OC43 showed similar effectivity against SARS-CoV-2; *n* = 3. Individual measurements are shown as semitransparent gray circles. (Note that some circles overlap.) Additional dose-response curves are shown in fig. S3.

We determined the EC_50_ values for 26 of these drugs against SARS-CoV-2 infection (excluding erythromycin, which failed validation, and tolertodine and imipramine, which were weak inhibitors of OC43 infection). In a high-biocontainment (BSL3) facility, human A549 cells overexpressing the angiotensin-converting enzyme 2 (ACE2) receptor were treated with the drugs for 2 hours, infected with SARS-CoV-2 (nCoV/Washington/1/2020) at an MOI of 0.5, incubated for 2 days, fixed, and stained for the viral spike protein (as a marker of SARS-CoV-2 infection). After staining, the cells were imaged, and the fraction of infected cells was quantified. Of the 26 drugs tested, 20 (77%) inhibited SARS-CoV-2 infection in a dose-dependent manner (Fig. 2 and fig. S3). Notably, the most potent drugs against OC43 infection (elbavir and amphotericin B) did not inhibit SARS-CoV-2 infection. A comparison of the EC_50_ values obtained against OC43 and SARS-CoV-2, as well as the chemical structures of the drugs, is shown in table S2. Thus, our screen identified 20 safe-in-human drugs that are able to inhibit both OC43 and SARS-CoV-2 infection of A549 cells.

**Fig. 3.**
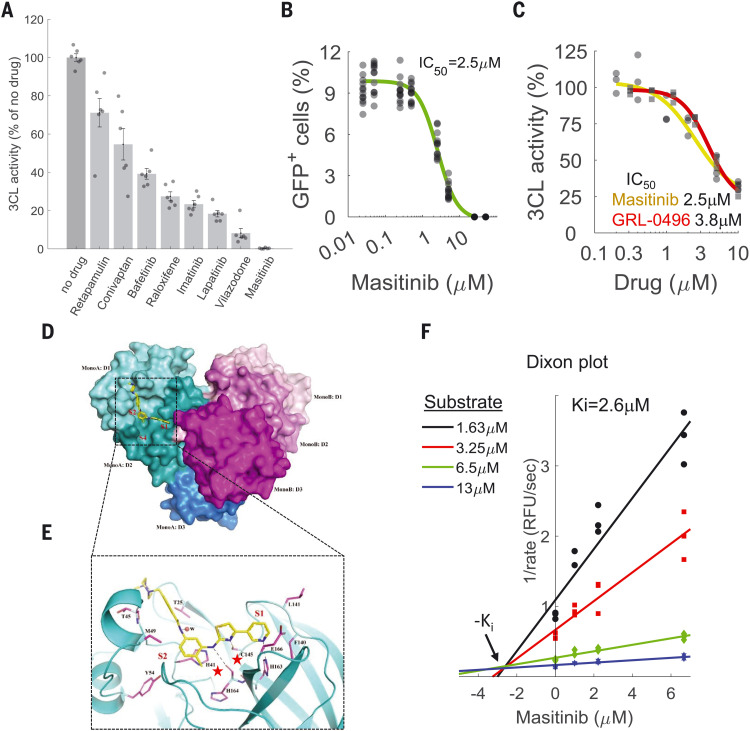
Masitinib inhibits SARS-CoV-2 3CLpro enzymatic activity. (**A**) A FlipGFP reporter assay was performed to screen for potential inhibition of 3CL by the identified drugs at a single concentration (10 μM). Shown are the drugs that showed a statistically significant reduction in 3CLpro activity (*p* < 0.05, one-tailed Student’s *t* test, false discovery rate corrected). *n* = 6. The data for the remaining tested drugs are shown in fig. S4. Individual measurements are shown as semitransparent gray circles. Error bars depict means ± SEM. (**B**) Dose-response curve for 3CL inhibition by masitinib using the FlipGFP reporter assay; *n* = 6. Individual measurements are shown as circles. (**C**) Dose-response curve for 3CL inhibition by masitinib (yellow) and GRL-0496 (red) using a luciferase reporter assay; *n* = 3. Individual measurements are shown as circles (masitinib) or squares (GRL-0496). (**D**) X-ray crystallography shows the dimer formation, domain structure, and masitinib binding site of SARS-CoV-2 3CL. Domains I, II, and III (D1 to D3) of the monomer A of a 3CL dimer are colored in cyan, teal, and light blue, respectively. The corresponding three domains of monomer B are colored in light pink, magenta, and purple. In monomer A, masitinib is drawn in stick format, bound to the active site. The locations of the three binding pockets S1, S2, and S4 are marked in red. (**E**) A ribbon diagram showing details of some interactions formed between masitinib and 3CL at the active site. Masitinib is drawn in stick format, with its C atoms colored in yellow. Key pocket-forming or interacting residues of 3CL are also presented in stick format, with their C atoms colored in purple. H-bonds are drawn as black dashed lines. The sites of binding pockets S1 and S2 are marked in red. The two catalytic residues are marked by red stars. The “w” represents a water molecule. C, Cys; E, Glu; F, Phe; H, His; L, Leu; M, Met; T, Thr; Y, Tyr. (**F**) Dixon plot, showing the rate of 3CL activity in the presence of different substrate and masitinib concentrations. *n* = 3. RFU, relative fluorescence units.

We next examined the ability of the drugs to inhibit the SARS-CoV-2 main protease, 3CL. 3CL is an attractive target for antiviral drugs because it is indispensable for viral replication and is well conserved among coronaviruses (*15*). Drugs that target 3CL are also unlikely to be affected by mutations that may arise in the spike protein owing to immunological pressure after natural infection or vaccination. We first tested the ability of the 20 drugs that inhibited both viruses to inhibit 3CL activity in human 293T cells transfected with a FlipGFP reporter system (*16*) at a single concentration of 10 μM. Eight drugs showed a statistically significant decrease in the percentage of green fluorescent protein (GFP)–expressing cells (Fig. 3A and fig. S4).

Most potent was masitinib, which completely inhibited 3CL activity in cells. Masitinib is an orally bioavailable c-kit inhibitor (*17*) that has been approved for treatment of mast cell tumors in dogs (*18*) and evaluated in phase 2 and 3 clinical trials in humans for the treatment of cancer (*19*), asthma (*20*), Alzheimer’s disease (*21*), multiple sclerosis (*22*), and amyotrophic lateral sclerosis (*23*). We determined the median inhibitory concentration (IC_50_) value (the drug concentration that causes a 50% reduction in enzymatic activity) of masitinib inhibition of 3CL activity in cells using two distinct assays: the FlipGFP reporter assay described above and a luciferase reporter assay (*24*). These assays, performed independently at the University of Chicago and Duke University, determined the IC_50_ value to be 2.5 μM (Fig. 3, B and C), similar to the EC_50_ values determined against OC43 (2.1 μM; Fig. 1C) and SARS-CoV-2 (3.2 μM; Fig. 2) infections, suggesting that masitinib inhibition of coronavirus infection is achieved by inhibiting 3CL activity. As a positive control, we determined the ability of GRL-0496, a covalent inhibitor of 3CL (*25*), to inhibit 3CL activity and found that it is similar to that of masitinib (Fig. 3C; IC_50_ = 3.8 μM), in agreement with its previously reported IC_50_ in cells (5 μM) (*26*). In agreement with this proposed mode of action (inhibition of viral replication after entry), masitinib was effective in inhibiting SARS-CoV-2 infection when added to cells 2 hours after infection (fig. S5A) and dramatically reduced viral progeny production by both wild-type and several of the main circulating variants of concern: B.1.1.7, B.1.351, and P.1 (fig. S5, B and C).

To obtain further mechanistic understanding of the mode of inhibition, we determined the high-resolution structure of masitinib-bound 3CL using x-ray crystallography (Fig. 3, D and E). The structure indicates that masitinib binds noncovalently between domains I and II of 3CL and blocks the key catalytic residues at the two active sites in the dimer.

Specifically, masitinib’s pyridine ring packs into the S1 peptide recognition site of 3CL (*27*). Besides hydrophobic and van der Waals interactions between the ring and its surrounding pocket-forming residues, the nitrogen atom of the pyridine forms a hydrogen bond (H-bond) (2.78 Å) with His^163^, located at the bottom of the S1 pocket. Masitinib’s aminothiazole ring forms two H-bonds with 3CL: one between its amine to His^164^ (3.09 Å) and one between the thiazole’s nitrogen to the Sγ atom of Cys^145^ (3.38 Å), the key catalytic residue of 3CL. The hydrophobic toluene ring of masitinib occupies the S2 binding pocket of the protease, forming a nearly perfect π-π stacking with His^41^, the second residue in the protease catalytic dyad. The benzamide group of masitinib points away from the S4 binding pocket of 3CL (which is used by the peptide substrate). Besides an H-bond from its amide to a nearby water molecule that is a part of a H-bond network, the benzamide mainly interacts with the main chain of the protease, between residues Cys^44^ and Ser^46^. The last portion of masitinib (the *N*-methylpiperaze group) is outside of the protease binding site and is disordered, with no corresponding electron densities in the Fourier maps (fig. S6).

Given that masitinib directly binds the catalytic residues of the protease (*28*), it likely acts as a competitive inhibitor. To test this, we measured the rate of the 3CL enzymatic reaction in vitro using a fluorescence-based enzyme activity assay (*29*). We measured the rate of 3CL activity in a range of substrate and inhibitor concentrations and fitted the data to equations describing different modes of inhibition using SigmaPlot Enzyme Kinetics Module 1.3 (see methods). As expected, competitive inhibition gave the best fit with the smallest Akaike information criterion corrections value. The inhibition constant, *K*_i_ (inhibitor concentration needed to occupy half of the enzyme active sites), was determined to be 2.6 μM, in good agreement with the IC_50_ values measured in the cellular assays (2.5 μM). In addition to the mathematical analysis of the data, competitive inhibition is also suggested from visualization of the data in a Dixon plot (*30*) (Fig. 3F), in which the convergence of the regression lines above the *x* axis is characteristic of competitive inhibition and their intersection denotes the *K*_i_ value.

Lastly, we evaluated the effect of masitinib on the activity of PLpro, the other viral protease, and found that it had no effect (fig. S7), supporting a specific role for masitinib in 3CL inhibition. Taken together, our results show that masitinib is a competitive inhibitor of 3CL that is able to bind to the active site of the enzyme and inhibit its catalytic activity, both in vitro and in live cells.

The 3C proteases of picornaviruses (human pathogens that cause a range of diseases, including the common cold, meningitis, hepatitis, and poliomyelitis) have extensive structural homology and share substrate specificity with 3CL (*31*). Using a luciferase reporter assay (*32*), we found that masitinib significantly inhibited the activity of the 3C protease (fig. S8A). Masitinib was also effective in blocking the replication of multiple picornaviruses (fig. S8B) but not the replication of other RNA viruses (fig. S8C). Thus, we conclude that masitinib is a relatively broad-spectrum antiviral that is able to inhibit multiple corona- and picornaviruses but not RNA viruses that do not rely on a 3CL-like protease to complete their life cycle.

Before evaluating the effectiveness of masitinib as an antiviral in vivo, we characterized the antiviral properties of its major metabolite, AB3280 (*33*). The structure of AB3280 is very similar to that of masitinib, missing only the terminal methyl group on the piperazine ring (which does not participate in binding 3CL; Fig. 3D). Indeed, we found that AB3280 maintains its antiviral activity against both OC43 and SARS-CoV-2 and binds to the 3CL active site in a similar manner (fig. S9).

**Fig. 4.**
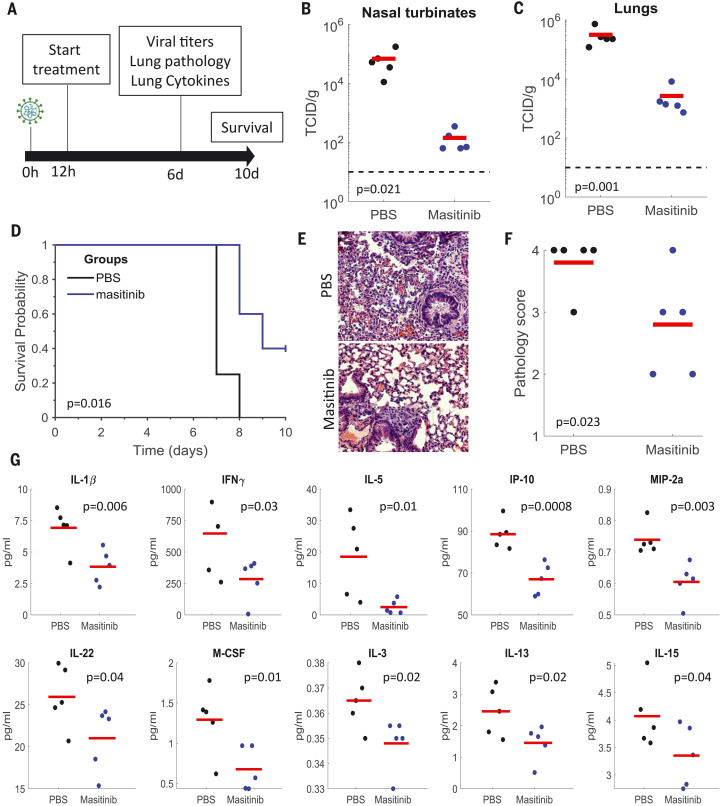
Masitinib inhibits SARS-CoV-2 replication in mice. (**A**) Schematic diagram of the experiment. (**B** and **C**) SARS-CoV-2 infectious virus measurements in the nose (B) and lungs (C) of PBS-treated (black) or masitinib-treated (blue) mice at day six after infection. *n* = 5 mice per group. Red lines are the mean values. The *p* values are shown in the figure (one-tailed Student’s *t* test). TCID, tissue culture infectious dose. (**D**) Kaplan-Meir curves assessing mice survival after PBS (black, *n* = 4) or masitinib (blue, *n* = 5) treatment after infection. The *p* value is shown in the figure (log-rank test). (**E**) Representative images of lung histology [hematoxylin and eosin (H&E)] stain at 6 days after infection. (**F**) Lung pathology score on day six after infection. Tissues were blindly scored on a scale of 0 to 4 by an expert veterinarian pathologist. *n* = 5 mice per group. The red lines are the mean values. The *p* value is shown in the figure (one-tailed Student’s *t* test). (**G**) Cytokine concentrations in the lungs of infected mice treated with PBS (black) or masitinib (blue) at day six after infection. *n* = 5 mice per group. The red lines are the mean values. The *p* values are shown in the figure (one-tailed Student’s *t* test). The experiment was performed once. IP-10, 10-kDa interferon gamma–induced protein; M-CSF, macrophage colony-stimulating factor 1; MIP-2a, macrophage inflammatory protein 2-alpha.

To evaluate the effect of masitinib on SARS-CoV-2 infection in mice, 20 K18-hACE2 transgenic mice (*34*) were intranasally infected with 2 × 10^4^ plaque-forming units of SARS-CoV-2 (nCoV/Washington/1/2020). Half were treated with phosphate-buffered saline (PBS) and half with masitinib [25 milligrams per kilogram of body weight (mg kg^−1^)] twice a day, starting 12 hours after infection and continuing for 10 days (Fig. 4A). This dose was well tolerated, with minimal weight loss in uninfected mice (fig. S10A). Twenty-five milligrams per kilogram is equivalent to 4 mg kg^−1^ day^−1^ in humans (*35*), within the safe doses used in human clinical trials. Most clinical trials in humans use 4.5 to 6 mg kg^−1^ day^−1^, with doses ranging up to 12 mg kg^−1^ day^−1^ (*36*). Five animals from each group were sacrificed on day six to assess viral loads and lung pathology, and the rest were used to analyze mice survival for up to 10 days (Fig. 4A). One mouse from the PBS-treated group was excluded from analysis because it showed no weight loss after infection (in contrast to the other 19 mice in the study).

Masitinib treatment resulted in a greater than two-log reduction in viral titers in the lungs and nose on day six (Fig. 4, B and C). It further improved overall lung pathology (as blindly assessed by a veterinarian pathologist; Fig. 4, E and F) and significantly reduced the concentrations of key proinflammatory cytokines [such as interleukin-1β (IL-1β) and interferon-γ (IFN-γ)] in the lungs (Fig. 4G). Further, we observed improvements in survival (Fig. 4D), weight loss (fig. S10B), and clinical scores (fig. S10C) with masitinib treatment. Taken together, our results show that masitinib is effective in reducing SARS-CoV-2 viral load in mice (reducing >99% of the viral load on day six), that it reduced inflammatory signatures, and that it showed potential benefits for survival and clinical scores.

In conclusion, we have shown that OC43, a BSL-2 pathogen that can be readily studied in most virological labs, is a good model system to screen for potential antiviral drugs against SARS-CoV-2 infection, because most drugs that inhibited OC43 replication also inhibited SARS-CoV-2 in our measurements. We identified 20 repurposed drugs that inhibited OC43 and SARS-CoV-2 replication and identified masitinib as an effective inhibitor of the viral protease 3CLpro.

Although the EC_50_ values for viral inhibition and the IC_50_ values for protease inhibition are in excellent agreement, we did not directly demonstrate that the inhibition of viral replication is the result of the inhibition of the protease activity. A direct way to test this is through the continuous propagation of the virus in the presence of low drug concentrations and the identification of escape mutants. Our attempts to recover such escape mutants with three different viruses (OC43, SARS-CoV-2, and CVB3) failed, suggesting a high barrier for acquiring resistance to the drug. Although an alternative explanation is that masitinib exerts its antiviral effect through inhibition of tyrosine kinases, two lines of evidence argue against it: First, our drug-repurposing screen included multiple other tyrosine kinase inhibitors that inhibit the same kinases with equal or better affinities than masitinib and that did not significantly inhibit OC43 infection (table S3). Second, multiple CRISPR-mediated screens showed that knockout of these tyrosine kinases did not affect SARS-CoV-2 and other coronavirus infections (*37*–*39*). Nevertheless, it is possible that the inhibition of one or more tyrosine kinases by masitinib contributes to its antiviral activity.

In addition to its direct antiviral effect described here, masitinib has been shown to decrease airway inflammation and improve lung functions in a feline model of asthma (*40*). Given that a main pathology of SARS-CoV-2 is acute respiratory distress syndrome, the combined antiviral and anti-inflammatory properties of masitinib might prove beneficial for treating COVID-19 patients. However, the timing of masitinib’s anti-inflammatory effects should be carefully studied, because it is not clear if a reduction in the inflammatory response would be desirable at the early phases of disease that are dominated by viral replication.

Future efforts should evaluate the efficacy of masitinib in treating COVID-19 patients. Although a phase 2 clinical trial has been registered with clinicaltrials.gov (identifier: NCT04622865) to test the effect of a combined treatment of masitinib and isoquercetin on hospitalized patients, our data suggest that masitinib would be most beneficial at early times after infection, when an antiviral is likely to have the biggest effect. Oral use of masitinib would make such early treatment feasible. Furthermore, future development of masitinib analogs with lower anti–tyrosine kinase activity would be beneficial to reduce its reported side effects. Masitinib is also interesting in that it is potent against multiple corona- and picornaviruses in vitro and may have potential for treating other viral diseases.

## Supplementary Material

20210720-1

## Supplementary Materials

science.sciencemag.org/content/373/6557/931/suppl/DC1
Materials and Methods Figs. S1 to S10 Tables S1 to S4 References (*41*–*54*) MDAR Reproducibility Checklist

## Appendix

View/request a protocol for this paper from *Bio-protocol*.
