# Supplementary material for: Masitinib is a broad coronavirus 3CL inhibitor that blocks replication of SARS-CoV-2
Source: Science. 2021 Jul 20;373(6557):931–6. doi: 10.1126/science.abg5827 (PMC8809056; doi:10.1126/science.abg5827)
Supplement: 20210720-1 [file science.abg5827.v1.pdf]

Cite as: N. Drayman *et al*., *Science* 10.1126/science.abg5827 (2021).

Masitinib is a broad coronavirus 3CL inhibitor that blocks replication of SARS-CoV-2

Nir Drayman[1]*, Jennifer K. DeMarco[2], Krysten A. Jones[3], Saara-Anne Azizi[3], Heather M. Froggatt[4], Kemin Tan[5,6], Natalia Ivanovna Maltseva[5,6], Siquan Chen[7], Vlad Nicolaescu[8], Steve Dvorkin[8], Kevin Furlong[8], Rahul S. Kathayat[3], Mason R. Firpo[9], Vincent Mastrodomenico[9], Emily A. Bruce[10,11], Madaline M. Schmidt[10,11], Robert Jedrzejczak[5,6], Miguel Á. Muñoz-Alía[12], Brooke Schuster[1], Vishnu Nair[1], Kyu-yeon Han[13], Amornrat O'Brien[9], Anastasia Tomatsidou[8], Bjoern Meyer[14], Marco Vignuzzi[14], Dominique Missiakas[8], Jason W. Botten[10,11,15], Christopher B. Brooke[16,17], Hyun Lee[18], Susan C. Baker[9], Bryan C. Mounce[9], Nicholas S. Heaton[4], William E. Severson[2], Kenneth E. Palmer[2], Bryan C. Dickinson[3], Andrzej Joachimiak[5,6,19], Glenn Randall[8], Savaş Tay[1]*

[1]The Pritzker School for Molecular Engineering, The University of Chicago, Chicago, IL, USA. [2]Center for Predictive Medicine for Biodefense and Emerging Infectious Diseases, University of Louisville, Louisville, KY, USA. [3]The Department of Chemistry, The University of Chicago, Chicago, IL, USA. [4]The Department of Molecular Genetics and Microbiology, Duke University, Durham, NC, USA. [5]Center for Structural Genomics of Infectious Diseases, Consortium for Advanced Science and Engineering, University of Chicago, Chicago, IL, USA. [6]Structural Biology Center, X-ray Science Division, Argonne National Laboratory, Argonne, IL, USA. [7]The Cellular Screening Center, The University of Chicago, Chicago, IL, USA. [8]Department of Microbiology, Ricketts Laboratory, University of Chicago, Chicago, IL, USA. [9]Department of Microbiology and Immunology, Stritch School of Medicine, Loyola University Chicago, Maywood, IL, USA. [10]Department of Medicine, Division of Immunobiology, Larner College of Medicine, University of Vermont, Burlington, VT, USA. [11]Department of Microbiology and Molecular Genetics, Larner College of Medicine, University of Vermont, Burlington, VT, USA. [12]Department of Molecular Medicine, Mayo Clinic, Rochester, MN, USA. [13]Department of Ophthalmology and Visual Sciences, Illinois Eye and Ear Infirmary, College of Medicine, University of Illinois at Chicago, Chicago, IL, USA. [14]Institut Pasteur, Viral Populations and Pathogenesis Unit, Centre National de la Recherche Scientifique UMR 3569, Paris, France. [15]Vaccine Testing Center, Larner College of Medicine, University of Vermont, Burlington, VT, USA. [16]Department of Microbiology, University of Illinois at Urbana-Champaign, Urbana, IL, USA. [17]Carl R. Woese Institute for Genomic Biology, University of Illinois at Urbana-Champaign, Urbana, IL, USA. [18]Department of Pharmaceutical Sciences, College of Pharmacy, Biophysics Core at Research Resources Center, University of Illinois at Chicago, Chicago, IL, USA. [19]The Department of Biochemistry and Molecular Biology, The University of Chicago, Chicago, IL, USA.

***Corresponding author. Email: tays@uchicago.edu (S.T.); nirdra@uchicago.edu (N.D.)**

There is an urgent need for antiviral agents that treat SARS-CoV-2 infection. We screened a library of 1,900 clinically safe drugs against OC43, a human beta-coronavirus that causes the common cold and evaluated the top hits against SARS-CoV-2. Twenty drugs significantly inhibited replication of both viruses in vitro. Eight of these drugs inhibited the activity of the SARS-CoV-2 main protease, 3CLpro, with the most potent being masitinib, an orally bioavailable tyrosine kinase inhibitor. X-ray crystallography and biochemistry show that masitinib acts as a competitive inhibitor of 3CLpro. Mice infected with SARS-CoV-2 and then treated with masitinib showed >200-fold reduction in viral titers in the lungs and nose, as well as reduced lung inflammation. Masitinib was also effective in vitro against all tested variants of concern (B.1.1.7, B.1.351 and P.1).

On January 2020, SARS-CoV-2 was identified as the causative agent of a new respiratory syndrome that was later named Corona Virus Disease 19 (COVID-19) (*1*). The virus has rapidly spread throughout the world, causing an ongoing pandemic, with millions of deaths (*2*). SARS-CoV-2 is a member of *Coronaviridae*, a family of enveloped, single-strand, positive-sense RNA viruses (*3*). This family is composed of both human and animal pathogens, including two other emerging human pathogens (SARS-CoV and MERS-CoV) as well four endemic human viruses that are the second most common cause of the common cold (HCoV-OC43, 229E, NL63 and HKU1) (*4*).

Upon entry into the host cell cytoplasm, the viral genome is translated into roughly 30 proteins. Of these, 16 are initially translated as two polyproteins that must be cleaved into the individual viral proteins for infection to proceed. This cleavage is mediated by two virally encoded proteases: the main viral protease, known as Mpro, 3CLpro or non-structural protein 5 (nsp5) and a second protease known as the papain-like protease, PLpro, a domain within nsp3 (*3*). There is interest in developing *de-novo* inhibitors to target these proteases (*5*–*10*) but this is a lengthy process.

While several vaccines received emergency use authorization from health authorities world-wide and are being deployed, it will take a long time to vaccinate the world population, and the emergence of viral escape mutants rendering vaccines ineffective remains a possibility. Therefore, there is continued need for new treatment options for COVID-19, as well as for broad-spectrum antivirals that could be used against future emerging viruses. Remdesivir, an

RNA-dependent RNA-polymerase inhibitor, has been reported to shorten COVID-19 hospitalization times (*11*), but it failed a large clinical trial in hospitalized patients (*12*) and its efficacy is unclear.

Drug-repurposing screens have been used to identify safe-in-human drugs with potential anti- SARS-CoV-2 properties (*9*, *13*, *14*). Repurposed drugs that have existing clinical data on the effective dose, treatment duration, side effects and toxicity information could be rapidly translated into the treatment of patients.

We screened a library of 1,900 clinically used drugs, either approved for human use or with extensive safety data in humans (Phase 2 or 3 clinical trials), for their ability to inhibit infection of A549 cells by OC43. We chose OC43 as it is a human pathogen that belongs to the same clade of beta-coronaviruses as SARS-CoV-2 and can be studied under "regular" biosafety conditions, as well as in an attempt to discover broad spectrum anti-coronavirus drugs that would be beneficial against SARS-CoV-2 and future emerging coronaviruses. One day after plating, cells were infected at an MOI of 0.3, incubated at 33°C for 1 hour and drugs were added to a final concentration of 10μM. Cells were then incubated at 33°C for 4 days, fixed and stained for the presence of the viral nucleoprotein (Fig. 1A). We imaged the cells at day 0 (following drug addition) and day 4 (after staining) to determine the drugs effect on cell growth and OC43 infection.

We repeated the screen twice and identified 108 drugs that significantly reduced OC43 infection (Fig. 1B and table S1). For further validation we looked at the top 35 hits, chose one drug in cases where it was tested in different formulations (such as Erythromycin Cyclocarbonate and Erythromycin estolate) and excluded drugs that were already evaluated against COVID19 and found ineffective (such as chloroquine) or that were withdrawn due to toxicity (such as Mesoridazine). We additionally included trimipramine, which was not present in our screen, because two closely related drugs (imipramine and clomipramine) were top hits. We determined the EC50 values (drug concentration required to reduce infection by 50%) of these 29 drugs against OC43 infection (Fig. 1C and fig. S1) as well as their effect on cell proliferation (CC50; fig. S2). With the exception of erythromycin, all drugs inhibited OC43 infection in a dose-dependent manner, with EC50 values ranging from 0.17-7μM.

We determined the EC50 values for 26 of these drugs against SARS-CoV-2 infection (excluding erythromycin that failed validation and tolertodine and imipramine that were weak inhibitors of OC43 infection). In a high biocontainment (BSL3) facility, A549 cells overexpressing the angiotensin-converting enzyme 2 (ACE2) receptor were treated with the drugs for 2 hours, infected with SARS-CoV-2 (nCoV/Washington/1/2020) at an MOI of 0.5, incubated for 2 days, fixed, and stained for the viral spike protein (as a marker of SARS-CoV-2 infection). After staining, the cells were imaged and the fraction of infected cells quantified. Of the 26 drugs tested, 20 (77%) inhibited SARS-CoV-2 infection in a dose dependent manner (Fig. 2 and fig. S3). Interestingly, the most potent drugs against OC43 infection (elbavir and amphotericin B) did not inhibit SARS-CoV-2 infection. A comparison of the EC50 values obtained against OC43 and SARS-CoV-2, as well as the chemical structures of the drugs, is shown in table S2. Thus, our screen identified 20 safe-in-human drugs that are able to inhibit both OC43 and SARS-CoV-2 infection of A549 cells.

We next examined the drugs ability to inhibit SARS-CoV-2 main protease, 3CL. 3CL is an attractive target for antiviral drugs, as it indispensable for viral replication and is well conserved among coronaviruses (*15*). Drugs that target 3CL are also unlikely to be affected by mutations that may arise in the Spike protein due to immunological pressure after natural infection or vaccination. We first tested the ability of the 20 drugs that inhibited both viruses to inhibit 3CL activity in 293T cells transfected with a FlipGFP reporter system (*16*) at a single concentration of 10μM. 8 drugs showed a statistically significant decrease in the percentage of GFP-expressing cells (Fig. 3A and fig. S4).

Most potent was masitinib, which completely inhibited 3CL activity in cells. Masitinib is an orally bioavailable c-kit inhibitor (*17*) that has been approved for treatment of mast-cell tumors in dogs (*18*) and evaluated in phase 2 and 3 clinical trials in humans for the treatment of cancer (*19*), asthma (*20*), Alzheimer's (*21*), multiple sclerosis (*22*) and amyotrophic lateral sclerosis (*23*).

We determined the IC50 value (the drug concentration that causes a 50% reduction in enzymatic activity) of masitinib inhibition of 3CL activity in cells using two distinct assays; the FlipGFP reporter assay described above and a luciferase reporter assay (*24*). These assays, performed independently at the University of Chicago and Duke University, determined the IC50 value to be 2.5μM (Fig. 3, B and C), similar to the EC50 values determined against OC43 (2.1μM; Fig. 1C) and SARS-CoV-2 (3.2μM; Fig. 2) infections, suggesting that masitinib inhibition of coronavirus infection is achieved by inhibiting 3CL activity. As a positive control, we determined the ability of GRL-0496, a covalent inhibitor of 3CL (*25*) to inhibit 3CL activity and found it is similar to that of masitinib (Fig. 3C; IC50=3.8μM), in agreement with its previously reported IC50 in cells (5μM) (*26*).

In agreement with this proposed mode-of-action (post-entry inhibition of viral replication), masitinib was effective in inhibiting SARS-CoV-2 infection when added to cells two hours after infection (fig. S5A) and dramatically reduced viral progeny production by both WT and several of the main circulating variants of concern; B.1.1.7, B.1.351 and P.1 (fig. S5, B and C).

To obtain further mechanistic understating of the mode of inhibition we determined the high resolution structure of masitinib-bound 3CL using X-ray crystallography (Fig. 3, D and E). The structure indicates that masitinib binds non-covalently between domains I and II of 3CL and blocks the key catalytic residues at the two active sites in the dimer.

Specifically, masitinib's pyridine ring packs into the S1 peptide recognition site of 3CL (*27*). Beside hydrophobic and Van der Waals interactions between the ring and its surrounding pocket-forming residues, the nitrogen atom of the pyridine forms an H-bond (2.78 Å) with His163, located at the bottom of the S1 pocket. Masitinib's aminothiazole ring forms two H-bonds with 3CL, one between its amine to His164 (3.09 Å) and one between the thiazole's nitrogen to the Sγ atom of Cys145 (3.38 Å), the key catalytic residue of 3CL. The hydrophobic toluene ring of masitinib occupies the S2 binding pocket of the protease, forming a nearly perfect π-π stacking with His41, the second residue in the protease catalytic dyad. The benzamide group of masitinib points away from the S4 binding pocket of 3CL (which is used by the peptide substrate). Besides an H-bond from its amide to a nearby water molecule that is a part of a hydrogen bond network, the benzamide mainly interacts with the mainchain of the protease, between residues Cys44-Ser46. The last portion of masitinib (the N-methylpiperaze group) is outside of the protease binding site and is disordered, with no corresponding electron densities in the Fourier maps (fig. S6).

Given that masitinib directly binds the catalytic residues of the protease (*28*), it likely acts as a competitive inhibitor. To test this, we measured the rate of 3CL enzymatic reaction in vitro using a fluorescence based enzyme activity assay (*29*). We measured the rate of 3CL activity in a range of substrate and inhibitor concentrations and fitted the data to equations describing different modes of inhibition using SigmaPlot Enzyme Kinetics Module 1.3 (see methods). As expected, competitive inhibition gave the best fit with the smallest Akaike Information Criterion corrections (AICc) value. The inhibition constant, K_i (inhibitor concentration needed to occupy half of the enzyme active sites) was determined to be 2.6μM, in good agreement with the IC50 values measured in the cellular assays (2.5μM). In addition to the mathematical analysis of the data, competitive inhibition is also suggested from its visualization in a Dixon plot (*30*) (Fig. 3F), in which the convergence of the regression lines above the x-axis is characteristic of competitive inhibition, and their intersection denotes the K_i value.

Lastly, we evaluated the effect of masitinib on the activity of PLpro, the other viral protease, and found it had no effect (fig. S7), supporting a specific role for masitinib in 3CL inhibition. Taken together, our results show that masitinib is a competitive inhibitor of 3CL, able to bind to the active site of the enzyme and inhibit its catalytic activity, both in vitro and in live cells.

The 3C proteases of picornaviruses (human pathogens that cause a range of diseases including the common cold, meningitis, hepatitis and poliomyelitis) have extensive structural homology and substrate specificity with 3CL (*31*). Using a luciferase reporter assay (*32*), we found that masitinib significantly inhibited the activity of the 3C protease (fig. S8A). Masitinib was also effective in blocking the replication of multiple picornaviruses (fig. S8B) but not of other RNA viruses (fig. S8C). Thus, we conclude that masitinib is a relatively broad-spectrum antiviral, able to inhibit multiple corona- and picorna- viruses, but not RNA viruses that do not rely on a 3CL-like protease to complete their life cycle.

Before evaluating the effectiveness of masitinib as an antiviral in vivo, we characterized the antiviral properties of its major metabolite, AB3280 (*33*). The structure of AB3280 is very similar to that of masitinib, missing only the terminal methyl group on the piperazine ring (which does not participate in binding 3CL; Fig. 3D). Indeed, we found that AB3280 maintains its antiviral activity against both OC43 and SARS-CoV-2, and binds to the 3CL active site in a similar manner (fig. S9).

To evaluate the effect of masitinib on SARS-CoV-2 infection in mice, twenty K18-hACE2 transgenic mice (*34*) were intranasally infected with $2X10^4$ PFU of SARS-CoV-2 (nCoV/Washington/1/2020). Half were treated with PBS and half with masitinib (25 mg/kg) twice a day, starting 12 hours post infection and followed for 10 days (Fig. 4A). This dose was well tolerated, with minimal weight loss in uninfected mice (fig. S10A). 25 mg/kg is equivalent to 4 mg/kg/day in humans (*35*), within the safe doses used in human clinical trials. Most clinical trials in humans use 4.5-6 mg/kg/day with doses ranging up to 12 mg/kg/day (*36*). 5 animals from each group were sacrificed on day 6 to assess viral loads and lung pathology and the rest were used to analyze mice survival for up to 10 days (Fig. 4A). One mouse from the PBS treated group was excluded from analysis, as it showed no weight loss following infection (in contrast to the other 19 mice in the study).

Masitinib treatment resulted in over 2-logs reduction in viral titers in the lungs and nose on day 6 (Fig. 4, B and C). It further improved overall lung pathology (as blindly assessed by a veterinarian pathologist; Fig. 4, E and F) and significantly reduced the levels of key pro-inflammatory cytokines (such as IL-1β and IFNγ) in the lungs (Fig. 4G). Further, we observed improvements in survival (Fig. 4D), weight loss (fig. S10B) and clinical scores (fig. S10C) with masitinib treatment. Taken together, our results show that masitinib is effective in reducing SARS-CoV-2 viral load in mice (reducing >99% of the viral load on day 6), reduced inflammatory signatures, and showed potential benefits for survival and clinical scores.

In conclusion, we have shown that OC43, a BSL-2

pathogen that can be readily studied in most virological labs, is a good model system to screen for potential antiviral drugs against SARS-CoV-2 infection, since most drugs that inhibited OC43 replication also inhibited SARS-CoV-2 in our measurements. We identified 20 repurposed drugs that inhibited OC43 and SARS-CoV-2 replication, and identified masitinib as a very effective inhibitor of the viral protease 3CLpro.

While the EC50 values for viral inhibition and the IC50 values for protease inhibition are in excellent agreement, we did not directly demonstrate that the inhibition of viral replication is the result of the inhibition of the protease activity. A direct way to test this is through the continuous propagation of the virus in the presence of low drug concentrations and the identification of escape mutants. Our attempts to recover such escape mutants with three different viruses (OC43, SARS-CoV-2 and CVB3) failed, suggesting a high barrier for acquiring resistance to the drug. While an alternative explanation is that masitinib exerts its antiviral effect through inhibition of tyrosine kinases, two lines of evidence argue against it: first, our drug-repurposing screen included multiple other tyrosine kinase inhibitors that inhibit the same kinases with equal or better affinities than masitinib and which did not significantly inhibit OC43 infection (table S3). Second, multiple CRISPR-mediated screens showed that knock-out of these tyrosine kinases did not affect SARS-CoV-2 and other coronaviruses infection (*37*–*39*). Nevertheless, it is possible that the inhibition of one or more tyrosine kinases by masitinib contributes to its antiviral activity.

In addition to its direct antiviral effect described here, masitinib has been shown to decrease airway inflammation and improve lung functions in a feline model of asthma (*40*). Given that a main pathology of SARS-CoV-2 is ARDS (acute respiratory distress syndrome), the combined antiviral and anti-inflammatory properties of masitinib might prove beneficial for treating COVID-19 patients. However, the timing of masitinib's anti-inflammatory effects should be carefully studied, as it is not clear if a reduction in the inflammatory response would be desirable at the early phases of disease that is dominated by viral replication.

Future efforts should evaluate the efficacy of masitinib in treating COVID-19 patients. While a phase 2 clinical trial has been registered with ClinicalTrials.gov (Identifier: NCT04622865) to test the effect of a combined treatment of masitinib and isoquercetin on hospitalized patients, our data suggests that masitinib would be most beneficial at early times after infection, when an antiviral is likely to have the biggest effect. Oral use of masitinib would make such early treatment feasible. Furthermore, future development of masitinib analogs with lower anti-tyrosine kinase activity would be beneficial to reduce its reported side effects. Masitinib is also interesting in that it is potent against multiple corona and picornaviruses in vitro and may have potential for treating other viral diseases.

REFERENCES AND NOTES

1. X. Yang, Y. Yu, J. Xu, H. Shu, J. Xia, H. Liu, Y. Wu, L. Zhang, Z. Yu, M. Fang, T. Yu, Y. Wang, S. Pan, X. Zou, S. Yuan, Y. Shang, Clinical course and outcomes of critically ill patients with SARS-CoV-2 pneumonia in Wuhan, China: A single-centered, retrospective, observational study. *Lancet Respir. Med.* **8**, 475–481 (2020). doi:10.1016/S2213-2600(20)30079-5 Medline
2. Worldometer, Coronavirus update (live), 22,559,115 cases and 790,019 deaths from COVID-19 virus pandemic; www.worldometers.info/coronavirus/.
3. Y. Chen, Q. Liu, D. Guo, Emerging coronaviruses: Genome structure, replication, and pathogenesis. *J. Med. Virol.* **92**, 418–423 (2020). doi:10.1002/jmv.25681 Medline
4. M. J. Mäkelä, T. Puhakka, O. Ruuskanen, M. Leinonen, P. Saikku, M. Kimpimäki, S. Blomqvist, T. Hyypiä, P. Arstila, Viruses and bacteria in the etiology of the common cold. *J. Clin. Microbiol.* **36**, 539–542 (1998). doi:10.1128/JCM.36.2.539-542.1998 Medline
5. Y. Liu, C. Liang, L. Xin, X. Ren, L. Tian, X. Ju, H. Li, Y. Wang, Q. Zhao, H. Liu, W. Cao, X. Xie, D. Zhang, Y. Wang, Y. Jian, The development of coronavirus 3C-like protease (3CL^{pro}) inhibitors from 2010 to 2020. *Eur. J. Med. Chem.* **206**, 112711 (2020). doi:10.1016/j.ejmech.2020.112711 Medline
6. W. Zhu, M. Xu, C. Z. Chen, H. Guo, M. Shen, X. Hu, P. Shinn, C. Klumpp-Thomas, S. G. Michael, W. Zheng, Identification of SARS-CoV-2 3CL protease inhibitors by a quantitative high-throughput screening. *ACS Pharmacol. Transl. Sci.* **3**, 1008–1016 (2020). doi:10.1021/acsptsci.0c00108 Medline
7. J. Osipiuk, S.-A. Azizi, S. Dvorkin, M. Endres, R. Jedrzejczak, K. A. Jones, S. Kang, R. S. Kathayat, Y. Kim, V. G. Lisnyak, S. L. Maki, V. Nicolaescu, C. A. Taylor, C. Tesar, Y.-A. Zhang, Z. Zhou, G. Randall, K. Michalska, S. A. Snyder, B. C. Dickinson, A. Joachimiak, Structure of papain-like protease from SARS-CoV-2 and its complexes with non-covalent inhibitors. *Nat. Commun.* **12**, 743 (2021). doi:10.1038/s41467-021-21060-3 Medline
8. W. Vuong, M. B. Khan, C. Fischer, E. Arutyunova, T. Lamer, J. Shields, H. A. Saffran, R. T. McKay, M. J. van Belkum, M. A. Joyce, H. S. Young, D. L. Tyrrell, J. C. Vederas, M. J. Lemieux, Feline coronavirus drug inhibits the main protease of SARS-CoV-2 and blocks virus replication. *Nat. Commun.* **11**, 4282 (2020). doi:10.1038/s41467-020-18096-2 Medline
9. Z. Jin, X. Du, Y. Xu, Y. Deng, M. Liu, Y. Zhao, B. Zhang, X. Li, L. Zhang, C. Peng, Y. Duan, J. Yu, L. Wang, K. Yang, F. Liu, R. Jiang, X. Yang, T. You, X. Liu, X. Yang, F. Bai, H. Liu, X. Liu, L. W. Guddat, W. Xu, G. Xiao, C. Qin, Z. Shi, H. Jiang, Z. Rao, H. Yang, Structure of M^{pro} from SARS-CoV-2 and discovery of its inhibitors. *Nature* **582**, 289–293 (2020). doi:10.1038/s41586-020-2223-y Medline
10. W. Dai, B. Zhang, X.-M. Jiang, H. Su, J. Li, Y. Zhao, X. Xie, Z. Jin, J. Peng, F. Liu, C. Li, Y. Li, F. Bai, H. Wang, X. Cheng, X. Cen, S. Hu, X. Yang, J. Wang, X. Liu, G. Xiao, H. Jiang, Z. Rao, L.-K. Zhang, Y. Xu, H. Yang, H. Liu, Structure-based design of antiviral drug candidates targeting the SARS-CoV-2 main protease. *Science* **368**, 1331–1335 (2020). doi:10.1126/science.abb4489 Medline
11. J. Pardo, A. M. Shukla, G. Chamarthi, A. Gupte, The journey of remdesivir: From Ebola to COVID-19. *Drugs Context* **9**, 1–9 (2020). doi:10.7573/dic.2020-4-14 Medline
12. J. H. Beigel, K. M. Tomashek, L. E. Dodd, A. K. Mehta, B. S. Zingman, A. C. Kalil, E. Hohmann, H. Y. Chu, A. Luetkemeyer, S. Kline, D. Lopez de Castilla, R. W. Finberg, K. Dierberg, V. Tapson, L. Hsieh, T. F. Patterson, R. Paredes, D. A. Sweeney, W. R. Short, G. Touloumi, D. C. Lye, N. Ohmagari, M.-D. Oh, G. M. Ruiz-Palacios, T. Benfield, G. Fätkenheuer, M. G. Kortepeter, R. L. Atmar, C. B. Creech, J. Lundgren, A. G. Babiker, S. Pett, J. D. Neaton, T. H. Burgess, T. Bonnett, M. Green, M. Makowski, A. Osinusi, S. Nayak, H. C. Lane, ACTT-1 Study Group Members, Remdesivir for the treatment of Covid-19—Final Report. *N. Engl. J. Med.* **383**, 1813–1826 (2020). doi:10.1056/NEJMoa2007764 Medline
13. D. E. Gordon, G. M. Jang, M. Bouhaddou, J. Xu, K. Obernier, K. M. White, M. J. O'Meara, V. V. Rezelj, J. Z. Guo, D. L. Swaney, T. A. Tummino, R. Hüttenhain, R. M. Kaake, A. L. Richards, B. Tutuncuoglu, H. Foussard, J. Batra, K. Haas, M. Modak, M. Kim, P. Haas, B. J. Polacco, H. Braberg, J. M. Fabius, M. Eckhardt, M. Soucheray, M. J. Bennett, M. Cakir, M. J. McGregor, Q. Li, B. Meyer, F. Roesch, T. Vallet, A. Mac Kain, L. Miorin, E. Moreno, Z. Z. C. Naing, Y. Zhou, S. Peng, Y. Shi, Z. Zhang, W. Shen, I. T. Kirby, J. E. Melnyk, J. S. Chorba, K. Lou, S. A. Dai, I. Barrio-Hernandez, D. Memon, C. Hernandez-Armenta, J. Lyu, C. J. P. Mathy, T. Perica, K.

B. Pilla, S. J. Ganesan, D. J. Saltzberg, R. Rakesh, X. Liu, S. B. Rosenthal, L. Calviello, S. Venkataramanan, J. Liboy-Lugo, Y. Lin, X.-P. Huang, Y. Liu, S. A. Wankowicz, M. Bohn, M. Safari, F. S. Ugur, C. Koh, N. S. Savar, Q. D. Tran, D. Shengjuler, S. J. Fletcher, M. C. O'Neal, Y. Cai, J. C. J. Chang, D. J. Broadhurst, S. Klippsten, P. P. Sharp, N. A. Wenzell, D. Kuzuoglu-Ozturk, H.-Y. Wang, R. Trenker, J. M. Young, D. A. Cavero, J. Hiatt, T. L. Roth, U. Rathore, A. Subramanian, J. Noack, M. Hubert, R. M. Stroud, A. D. Frankel, O. S. Rosenberg, K. A. Verba, D. A. Agard, M. Ott, M. Emerman, N. Jura, M. von Zastrow, E. Verdin, A. Ashworth, O. Schwartz, C. d'Enfert, S. Mukherjee, M. Jacobson, H. S. Malik, D. G. Fujimori, T. Ideker, C. S. Craik, S. N. Floor, J. S. Fraser, J. D. Gross, A. Sali, B. L. Roth, D. Ruggero, J. Taunton, T. Kortemme, P. Beltrao, M. Vignuzzi, A. García-Sastre, K. M. Shokat, B. K. Shoichet, N. J. Krogan, A SARS-CoV-2 protein interaction map reveals targets for drug repurposing. *Nature* **583**, 459–468 (2020). doi:10.1038/s41586-020-2286-9 Medline

14. L. Riva, S. Yuan, X. Yin, L. Martin-Sancho, N. Matsunaga, L. Pache, S. Burgstaller-Muehlbacher, P. D. De Jesus, P. Teriete, M. V. Hull, M. W. Chang, J. F.-W. Chan, J. Cao, V. K.-M. Poon, K. M. Herbert, K. Cheng, T. H. Nguyen, A. Rubanov, Y. Pu, C. Nguyen, A. Choi, R. Rathnasinghe, M. Schotsaert, L. Miorin, M. Dejosez, T. P. Zwaka, K.-Y. Sit, L. Martinez-Sobrido, W.-C. Liu, K. M. White, M. E. Chapman, E. K. Lendy, R. J. Glynne, R. Albrecht, E. Ruppin, A. D. Mesecar, J. R. Johnson, C. Benner, R. Sun, P. G. Schultz, A. I. Su, A. García-Sastre, A. K. Chatterjee, K.-Y. Yuen, S. K. Chanda, Discovery of SARS-CoV-2 antiviral drugs through large-scale compound repurposing. *Nature* **586**, 113–119 (2020). doi:10.1038/s41586-020-2577-1 Medline
15. K. Anand, J. Ziebuhr, P. Wadhwani, J. R. Mesters, R. Hilgenfeld, Coronavirus main proteinase (3CLpro) structure: Basis for design of anti-SARS drugs. *Science* **300**, 1763–1767 (2003). doi:10.1126/science.1085658 Medline
16. H. M. Froggatt, B. E. Heaton, N. S. Heaton, Development of a fluorescence based, high-throughput SARS-CoV-2 3CL^{pro} reporter assay. *J. Virol.* **94**, e01265–e20 (2020). doi:10.1128/JVI.01265-20 Medline
17. P. Dubreuil, S. Letard, M. Ciufolini, L. Gros, M. Humbert, N. Castéran, L. Borge, B. Hajem, A. Lermet, W. Sippl, E. Voisset, M. Arock, C. Auclair, P. S. Leventhal, C. D. Mansfield, A. Moussy, O. Hermine, Masitinib (AB1010), a potent and selective tyrosine kinase inhibitor targeting KIT. *PLOS ONE* **4**, e7258 (2009). doi:10.1371/journal.pone.0007258 Medline
18. K. A. Hahn, G. Ogilvie, T. Rusk, P. Devauchelle, A. Leblanc, A. Legendre, B. Powers, P. S. Leventhal, J.-P. Kinet, F. Palmerini, P. Dubreuil, A. Moussy, O. Hermine, Masitinib is safe and effective for the treatment of canine mast cell tumors. *J. Vet. Intern. Med.* **22**, 1301–1309 (2008). doi:10.1111/j.1939-1676.2008.0190.x Medline
19. A. Ottaiano, M. Capozzi, C. De Divitiis, A. De Stefano, G. Botti, A. Avallone, S. Tafuto, Gemcitabine mono-therapy versus gemcitabine plus targeted therapy in advanced pancreatic cancer: A meta-analysis of randomized phase III trials. *Acta Oncol.* **56**, 377–383 (2017). doi:10.1080/0284186X.2017.1288922 Medline
20. M. Humbert, F. de Blay, G. Garcia, A. Prud'homme, C. Leroyer, A. Magnan, J.-M. Tunon-de-Lara, C. Pison, M. Aubier, D. Charpin, I. Vachier, A. Purohit, P. Gineste, T. Bader, A. Moussy, O. Hermine, P. Chanez, Masitinib, a c-kit/PDGF receptor tyrosine kinase inhibitor, improves disease control in severe corticosteroid-dependent asthmatics. *Allergy* **64**, 1194–1201 (2009). doi:10.1111/j.1398-9995.2009.02122.x Medline
21. J. Folch, D. Petrov, M. Ettcheto, I. Pedrós, S. Abad, C. Beas-Zarate, A. Lazarowski, M. Marin, J. Olloquequi, C. Auladell, A. Camins, Masitinib for the treatment of mild to moderate Alzheimer's disease. *Expert Rev. Neurother.* **15**, 587–596 (2015). doi:10.1586/14737175.2015.1045419 Medline
22. P. Vermersch, R. Benrabah, N. Schmidt, H. Zéphir, P. Clavelou, C. Vongsouthi, P. Dubreuil, A. Moussy, O. Hermine, Masitinib treatment in patients with progressive multiple sclerosis: A randomized pilot study. *BMC Neurol.* **12**, 36 (2012). doi:10.1186/1471-2377-12-36 Medline
23. J. S. Mora, A. Genge, A. Chio, C. J. Estol, D. Chaverri, M. Hernández, S. Marín, J. Mascias, G. E. Rodriguez, M. Povedano, A. Paipa, R. Dominguez, J. Gamez, M. Salvado, C. Lunetta, C. Ballario, N. Riva, J. Mandrioli, A. Moussy, J.-P. Kinet, C. Auclair, P. Dubreuil, V. Arnold, C. D. Mansfield, O. Hermine, AB10015 Study Group, Masitinib as an add-on therapy to riluzole in patients with amyotrophic lateral sclerosis: A randomized clinical trial. *Amyotroph. Lateral Scler. Frontotemporal Degener.* **21**, 5–14 (2020). doi:10.1080/21678421.2019.1632346 Medline
24. A. O'Brien, D.-Y. Chen, M. Hackbart, B. J. Close, T. E. O'Brien, M. Saeed, S. C. Baker, Detecting SARS-CoV-2 3CLpro expression and activity using a polyclonal antiserum and a luciferase-based biosensor. *Virology* **556**, 73–78 (2021). doi:10.1016/j.virol.2021.01.010 Medline
25. A. K. Ghosh, G. Gong, V. Grum-Tokars, D. C. Mulhearn, S. C. Baker, M. Coughlin, B. S. Prabhakar, K. Sleeman, M. E. Johnson, A. D. Mesecar, Design, synthesis and antiviral efficacy of a series of potent chloropyridyl ester-derived SARS-CoV 3CLpro inhibitors. *Bioorg. Med. Chem. Lett.* **18**, 5684–5688 (2008). doi:10.1016/j.bmcl.2008.08.082 Medline
26. S. J. Resnick, S. Iketani, S. J. Hong, A. Zask, H. Liu, S. Kim, S. Melore, M. S. Nair, Y. Huang, N. E. S. Tay, T. Rovis, H. W. Yang, B. R. Stockwell, D. D. Ho, A. Chavez, A simplified cell-based assay to identify coronavirus 3CL protease inhibitors. bioRxiv 2020.08.29.272864 (2020); https://doi.org/10.1101/2020.08.29.272864.
27. K. Anand, G. J. Palm, J. R. Mesters, S. G. Siddell, J. Ziebuhr, R. Hilgenfeld, Structure of coronavirus main proteinase reveals combination of a chymotrypsin fold with an extra alpha-helical domain. *EMBO J.* **21**, 3213–3224 (2002). doi:10.1093/emboj/cdf327 Medline
28. D. W. Kneller, G. Phillips, H. M. O'Neill, R. Jedrzejczak, L. Stols, P. Langan, A. Joachimiak, L. Coates, A. Kovalevsky, Structural plasticity of SARS-CoV-2 3CL M^{pro} active site cavity revealed by room temperature X-ray crystallography. *Nat. Commun.* **11**, 3202 (2020). doi:10.1038/s41467-020-16954-7 Medline
29. H. Lee, A. Mittal, K. Patel, J. L. Gatuz, L. Truong, J. Torres, D. C. Mulhearn, M. E. Johnson, Identification of novel drug scaffolds for inhibition of SARS-CoV 3-Chymotrypsin-like protease using virtual and high-throughput screenings. *Bioorg. Med. Chem.* **22**, 167–177 (2014). doi:10.1016/j.bmc.2013.11.041 Medline
30. M. Dixon, The determination of enzyme inhibitor constants. *Biochem. J.* **55**, 170–171 (1953). doi:10.1042/bj0550170 Medline
31. C.-C. Lee, C.-J. Kuo, T.-P. Ko, M.-F. Hsu, Y.-C. Tsui, S.-C. Chang, S. Yang, S.-J. Chen, H.-C. Chen, M.-C. Hsu, S.-R. Shih, P.-H. Liang, A. H.-J. Wang, Structural basis of inhibition specificities of 3C and 3C-like proteases by zinc-coordinating and peptidomimetic compounds. *J. Biol. Chem.* **284**, 7646–7655 (2009). doi:10.1074/jbc.M807947200 Medline
32. C. N. Dial, P. M. Tate, T. M. Kicmal, B. C. Mounce, Coxsackievirus B3 responds to polyamine depletion via enhancement of 2A and 3C protease activity. *Viruses* **11**, 403 (2019). doi:10.3390/v11050403 Medline
33. I. Marech, R. Patruno, N. Zizzo, C. Gadaleta, M. Introna, A. F. Zito, C. D. Gadaleta, G. Ranieri, Masitinib (AB1010), from canine tumor model to human clinical development: Where we are? *Crit. Rev. Oncol. Hematol.* **91**, 98–111 (2014). doi:10.1016/j.critrevonc.2013.12.011 Medline
34. P. B. McCray Jr., L. Pewe, C. Wohlford-Lenane, M. Hickey, L. Manzel, L. Shi, J. Netland, H. P. Jia, C. Halabi, C. D. Sigmund, D. K. Meyerholz, P. Kirby, D. C. Look, S. Perlman, Lethal infection of K18-hACE2 mice infected with severe acute respiratory syndrome coronavirus. *J. Virol.* **81**, 813–821 (2007). doi:10.1128/JVI.02012-06 Medline
35. U.S. Department of Health and Human Services, Guidance for industry on estimating the maximum safe starting dose in initial clinical trials for therapeutics in adult healthy volunteers, availability. *Fed. Reg.* **70**, 42346 (2005); www.federalregister.gov/documents/2005/07/22/05-14456/guidance-for-industry-on-estimating-the-maximum-safe-starting-dose-in-initial-clinical-trials-for.
36. J. C. Soria, C. Massard, N. Magné, T. Bader, C. D. Mansfield, J. Y. Blay, B. N. Bui, A. Moussy, O. Hermine, J. P. Armand, Phase 1 dose-escalation study of oral tyrosine kinase inhibitor masitinib in advanced and/or metastatic solid cancers. *Eur. J. Cancer* **45**, 2333–2341 (2009). doi:10.1016/j.ejca.2009.05.010 Medline
37. W. M. Schneider, J. M. Luna, H.-H. Hoffmann, F. J. Sánchez-Rivera, A. A. Leal, A. W. Ashbrook, J. Le Pen, I. Ricardo-Lax, E. Michailidis, A. Peace, A. F. Stenzel, S. W. Lowe, M. R. MacDonald, C. M. Rice, J. T. Poirier, Genome-scale identification of SARS-CoV-2 and pan-coronavirus host factor networks. *Cell* **184**, 120–132.e14 (2021). doi:10.1016/j.cell.2020.12.006 Medline
38. J. Baggen, L. Persoons, E. Vanstreels, S. Jansen, D. Van Looveren, B. Boeckx, V. Geudens, J. De Man, D. Jochmans, J. Wauters, E. Wauters, B. M. Vanaudenaerde, D. Lambrechts, J. Neyts, K. Dallmeier, H. J. Thibaut, M. Jacquemyn, P. Maes, D. Daelemans, Genome-wide CRISPR screening identifies TMEM106B as a proviral host factor for SARS-CoV-2. *Nat. Genet.* **53**, 435–444 (2021). doi:10.1038/s41588-021-00805-2 Medline

39. Y. Zhu, F. Feng, G. Hu, Y. Wang, Y. Yu, Y. Zhu, W. Xu, X. Cai, Z. Sun, W. Han, R. Ye, D. Qu, Q. Ding, X. Huang, H. Chen, W. Xu, Y. Xie, Q. Cai, Z. Yuan, R. Zhang, A genome-wide CRISPR screen identifies host factors that regulate SARS-CoV-2 entry. *Nat. Commun.* **12**, 961 (2021). doi:10.1038/s41467-021-21213-4 Medline
40. T. M. Lee-Fowler, V. Guntur, J. Dodam, L. A. Cohn, A. E. DeClue, C. R. Reinero, The tyrosine kinase inhibitor masitinib blunts airway inflammation and improves associated lung mechanics in a feline model of chronic allergic asthma. *Int. Arch. Allergy Immunol.* **158**, 369–374 (2012). doi:10.1159/000335122 Medline
41. D. Blanco-Melo, B. E. Nilsson-Payant, W.-C. Liu, S. Uhl, D. Hoagland, R. Møller, T. X. Jordan, K. Oishi, M. Panis, D. Sachs, T. T. Wang, R. E. Schwartz, J. K. Lim, R. A. Albrecht, B. R. tenOever, Imbalanced host response to SARS-CoV-2 drives development of COVID-19. *Cell* **181**, 1036–1045.e9 (2020). doi:10.1016/j.cell.2020.04.026 Medline
42. L. Flatz, A. Bergthaler, J. C. de la Torre, D. D. Pinschewer, Recovery of an arenavirus entirely from RNA polymerase I/II-driven cDNA. *Proc. Natl. Acad. Sci. U.S.A.* **103**, 4663–4668 (2006). doi:10.1073/pnas.0600652103 Medline
43. C. M. Ziegler, P. Eisenhauer, E. A. Bruce, M. E. Weir, B. R. King, J. P. Klaus, D. N. Krementsov, D. J. Shirley, B. A. Ballif, J. Botten, The lymphocytic choriomeningitis virus matrix protein PPXY late domain drives the production of defective interfering particles. *PLOS Pathog.* **12**, e1005501 (2016). doi:10.1371/journal.ppat.1005501 Medline
44. J. R. del Valle, P. Devaux, G. Hodge, N. J. Wegner, M. B. McChesney, R. Cattaneo, A vectored measles virus induces hepatitis B surface antigen antibodies while protecting macaques against measles virus challenge. *J. Virol.* **81**, 10597–10605 (2007). doi:10.1128/JVI.00923-07 Medline
45. M. A. Muñoz-Alía, S. J. Russell, Probing morbillivirus antisera neutralization using functional chimerism between measles virus and canine distemper virus envelope glycoproteins. *Viruses* **11**, 688 (2019). doi:10.3390/v11080688 Medline
46. X. Xue, H. Yang, W. Shen, Q. Zhao, J. Li, K. Yang, C. Chen, Y. Jin, M. Bartlam, Z. Rao, Production of authentic SARS-CoV M(pro) with enhanced activity: Application as a novel tag-cleavage endopeptidase for protein overproduction. *J. Mol. Biol.* **366**, 965–975 (2007). doi:10.1016/j.jmb.2006.11.073 Medline
47. Y. Kim, G. Babnigg, R. Jedrzejczak, W. H. Eschenfeldt, H. Li, N. Maltseva, C. Hatzos-Skintges, M. Gu, M. Makowska-Grzyska, R. Wu, H. An, G. Chhor, A. Joachimiak, High-throughput protein purification and quality assessment for crystallization. *Methods* **55**, 12–28 (2011). doi:10.1016/j.ymeth.2011.07.010 Medline
48. W. Minor, M. Cymborowski, Z. Otwinowski, M. Chruszcz, HKL-3000: The integration of data reduction and structure solution—from diffraction images to an initial model in minutes. *Acta Crystallogr. D Biol. Crystallogr.* **62**, 859–866 (2006). doi:10.1107/S0907444906019949 Medline
49. A. Vagin, A. Teplyakov, Molecular replacement with MOLREP. *Acta Crystallogr. D Biol. Crystallogr.* **66**, 22–25 (2010). doi:10.1107/S0907444909042589 Medline
50. P. Emsley, K. Cowtan, Coot: Model-building tools for molecular graphics. *Acta Crystallogr. D Biol. Crystallogr.* **60**, 2126–2132 (2004). doi:10.1107/S0907444904019158 Medline
51. T. C. Terwilliger, R. J. Read, P. D. Adams, A. T. Brunger, P. V. Afonine, R. W. Grosse-Kunstleve, L.-W. Hung, Improved crystallographic models through iterated local density-guided model deformation and reciprocal-space refinement. *Acta Crystallogr. D Biol. Crystallogr.* **68**, 861–870 (2012). doi:10.1107/S0907444912015636 Medline
52. V. B. Chen, W. B. Arendall III, J. J. Headd, D. A. Keedy, R. M. Immormino, G. J. Kapral, L. W. Murray, J. S. Richardson, D. C. Richardson, MolProbity: All-atom structure validation for macromolecular crystallography. *Acta Crystallogr. D Biol. Crystallogr.* **66**, 12–21 (2010). doi:10.1107/S0907444909042073 Medline
53. N. R. Graham, A. N. Whitaker, C. A. Strother, A. K. Miles, D. Grier, B. D. McElvany, E. A. Bruce, M. E. Poynter, K. K. Pierce, B. D. Kirkpatrick, R. D. Stapleton, G. An, J. W. Botten, J. W. Crothers, S. A. Diehl, Kinetics and isotype assessment of antibodies targeting the spike protein receptor binding domain of SARS-CoV-2 in COVID-19 patients as a function of age and biological sex. medRxiv 2020.07.15.20154443 (2010); https://doi.org/10.1101/2020.07.15.20154443.
54. C. M. Ziegler, P. Eisenhauer, E. A. Bruce, V. Beganovic, B. R. King, M. E. Weir, B. A. Ballif, J. Botten, A novel phosphoserine motif in the LCMV matrix protein Z regulates the release of infectious virus and defective interfering particles. *J. Gen. Virol.* **97**, 2084–2089 (2016). doi:10.1099/jgv.0.000550 Medline

ACKNOWLEDGMENTS

We thank the members of the SBC at Argonne National Laboratory, especially Darren Sherrell and Alex Lavens for their help with setting beamline and data collection at beamline 19-ID. We would like to thank Lucas F. Welk for assistance in purification of 3CLpro. The rLCMV Armstrong 53b reverse genetics system was generously provided by Juan Carlos de la Torre. We thank AB Science for supplying masitinib for in vivo studies and the active metabolite of masitinib, AB3280. **Funding:** ND is a recipient of the Human Frontiers Science Program (HFSP) post-doctoral fellowship. Funding for this project was provided in part by federal funds from the National Institute of Allergy and Infectious Diseases, National Institutes of Health, Department of Health and Human Services, under Contract HHSN272201700060C (to AJ) and by the DOE Office of Science through the National Virtual Biotechnology Laboratory, a consortium of DOE national laboratories focused on response to COVID-19, with funding provided by the Coronavirus CARES Act (to AJ). EAB acknowledges funding by NIH P20GM125498 (UVM Translational Global Infectious Disease Research Center) and NIH P30GM118228-04 (UVM Center for Immunology and Infectious Disease). JWB acknowledges funding from the Office of the Vice President for Research at the University of Vermont and NIH grants R41AI132047, R21AI154198, and U01AI1141997. BM acknowledges the NIGMS grant R35GM138199. The use of SBC beamlines at the Advanced Photon Source is supported by the U.S. Department of Energy (DOE) Office of Science and operated for the DOE Office of Science by Argonne National Laboratory under Contract No. DE-AC02-06CH11357. BD acknowledges funding from the National Institute of General Medical Sciences (R35 GM119840). KP acknowledges funding from the NIH grant P20 GM125504. SCB acknowledges funding from NIH grant R01 AI085089. ST acknowledges funding support by the Pritzker School of Molecular Engineering at The University of Chicago. **Author contributions:** ND conducted the OC43 drug screening and dose-response experiments, analyzed data, generated figures, wrote the manuscript and supervised the project. JKM Performed all in vivo mouse work and downstream assays. SAA, KAJ, HMF, KYH, AO and HL performed experiments for determining 3CL inhibition. KT and NM conducted and analyzed the x-ray crystallography and generated figures. SC conducted the OC43 drug screening and dose-response experiments. VN, SD and KF conducted and analyzed the SARS-CoV-2 dose response and tittering experiments. MRF, VM and BCM conducted and analyzed the picornaviruses experiments. EAB, MMS and JWB conducted and analyzed the LCMV experiments. RJ designed the recombinant 3CLpro purification system. MMA conducted and analyzed the Measles experiments. BS and VN assisted in data analysis. CBB conducted and analyzed the IAV experiments. KEP and WES reviewed data and contributed to study design/implementation. SCB supervised and designed the 3CL luciferase assay and wrote the manuscript. NSH supervised the 3CL FLipGFP assay and wrote the manuscript. BCD supervised the 3CL kinetics assay and luciferase assay and wrote the manuscript. AJ supervised the x-ray crystallography and wrote the manuscript. GR supervised the SARS-CoV-2 experiments and wrote the manuscript. ST supervised the project and wrote the manuscript. AT and DM helped analyze the in vivo data and revise the manuscript. BM conducted experiments with SARS-CoV-2 variants of concern. MV supervised experiments with SARS-CoV-2 variants of concern and helped revise the manuscript. **Competing interests:** The University of Chicago has filed patent applications related to the work described herein (US patents No. 63/062,775 and No. 63/127,436). The University of Chicago and AB Science filed an international patent application related to the work described herein (No. PCT/EP2021/059355). ND, ST, GR and SC are listed as inventors on these applications. The University of Chicago has recently started an active sponsored research agreement to develop and test novel antiviral drugs with AB Science. **Data and materials availability**: X-ray crystallography data has been deposited in the Protein Data Bank (PDB) and is freely available under accession numbers 7JU7 and 7L5D. All other data are available in the manuscript or the supplementary material. The FlipGFP system is available from Dr. Nicholas Heaton under a material transfer agreement with Duke University. SARS-CoV-2 (nCoV/Washington/1/2020) is available form Natalia Thornburg (Centers for Disease Control) via the World Reference Center for Emerging Viruses and Arboviruses (Galveston, Texas). This work is licensed under a Creative Commons Attribution 4.0 International (CC BY 4.0) license,

which permits unrestricted use, distribution, and reproduction in any medium, provided the original work is properly cited. To view a copy of this license, visit https://creativecommons.org/licenses/by/4.0/. This license does not apply to figures/photos/artwork or other content included in the article that is credited to a third party; obtain authorization from the rights holder before using such material.

SUPPLEMENTARY MATERIALS

science.sciencemag.org/cgi/content/full/science.abg5827/DC1
Materials and Methods
Figs. S1 to S10
Tables S1 to S4
References (*41*–*54*)

14 January 2021; accepted 14 July 2021
Published online 20 July 2021
10.1126/science.abg5827

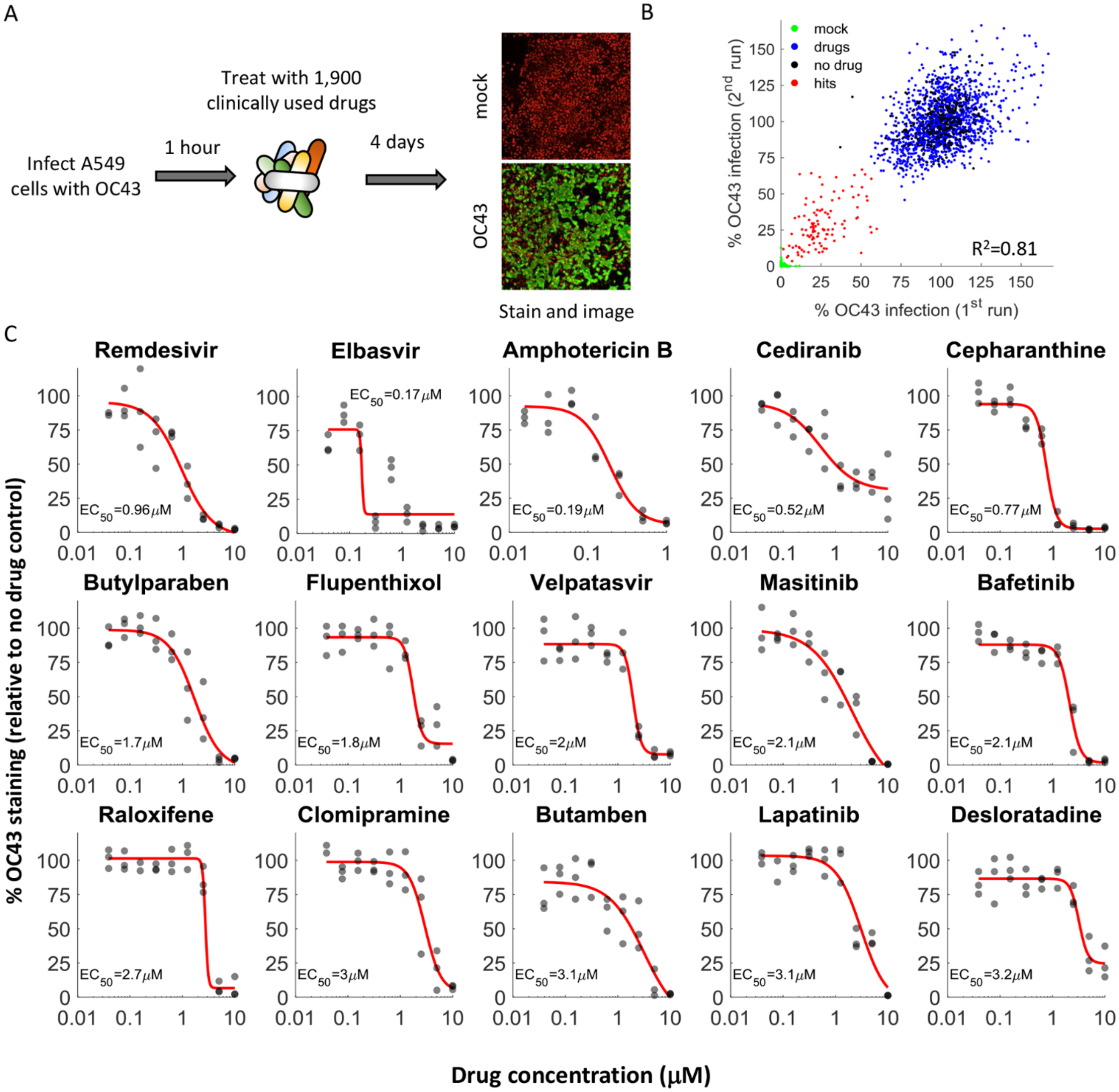

Fig. 1. A drug repurposing screen identifies multiple safe-in-human drugs that inhibit OC43 infection. (**A**) Schematic of the screen. A549 cells expressing H2B-mRuby were infected with OC43 (MOI 0.3), treated with drugs, incubated for 4 days at 33°C, and stained for the viral nucleoprotein. (**B**) Screen results showing the %OC43 staining of mock-infected cells (green), no-drug controls (black), drugs with no effect on OC43 infection (blue), and screen hits (red). Overall agreement between the two repeats is high (R^2=0.81) (**C**) Dose response curves of remdesivir and the top hits from the screen, n = 3. Individual measurements are shown as semi-transparent circles (note that some circles overlap). Additional dose response curves are shown in fig. S1.

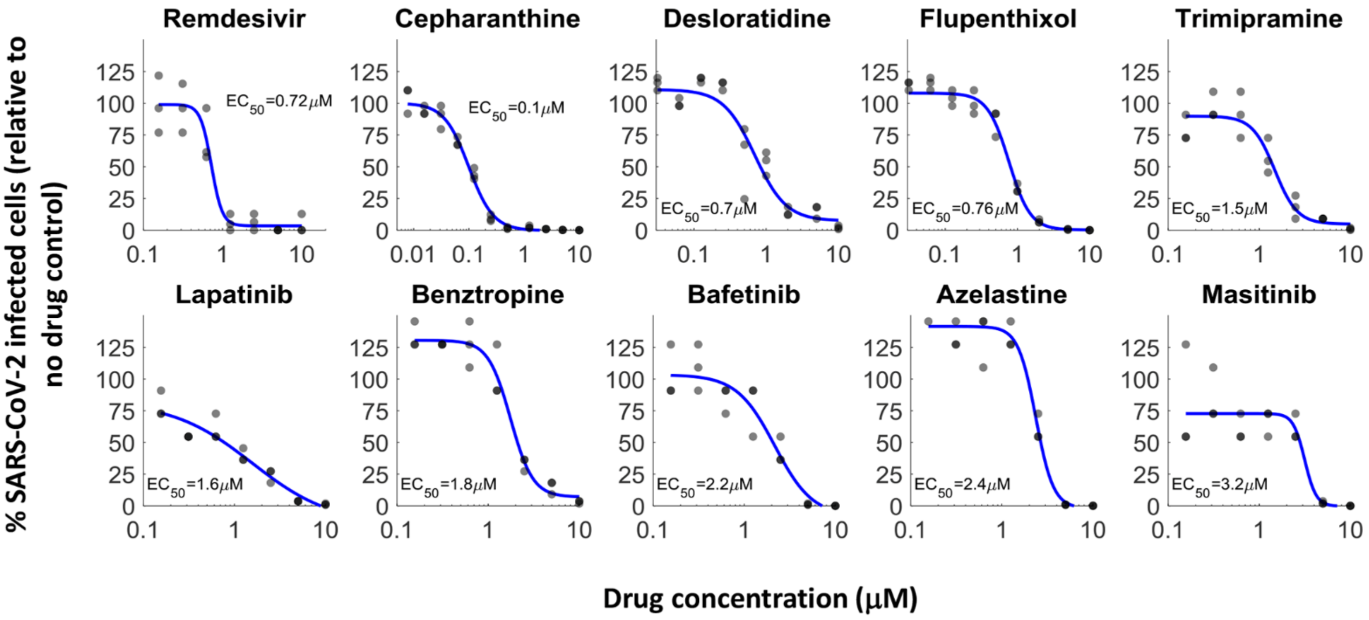

Fig. 2. Discovery of repurposed drugs that inhibit SARS-CoV-2 in human lung cells. Of the 26 drugs that inhibited OC43 and tested against SARS-CoV-2, 20 inhibited SARS-CoV-2 replication in a dose-dependent manner, showing good concordance between OC43 and SARS-CoV-2 inhibition. A549 cells overexpressing ACE2 were pre-treated with indicated drugs for 2 hours, infected with SARS-CoV-2 (MOI 0.5) and incubated for 2 days. Cells were stained for the presence of the spike protein and the % of infected cells was analyzed. Most of the drugs effective against OC43 showed similar effectivity against SARS-CoV-2, n=3. Individual measurements are shown as semi-transparent circles (note that some circles overlap). Additional dose response curves are shown in fig. S3.

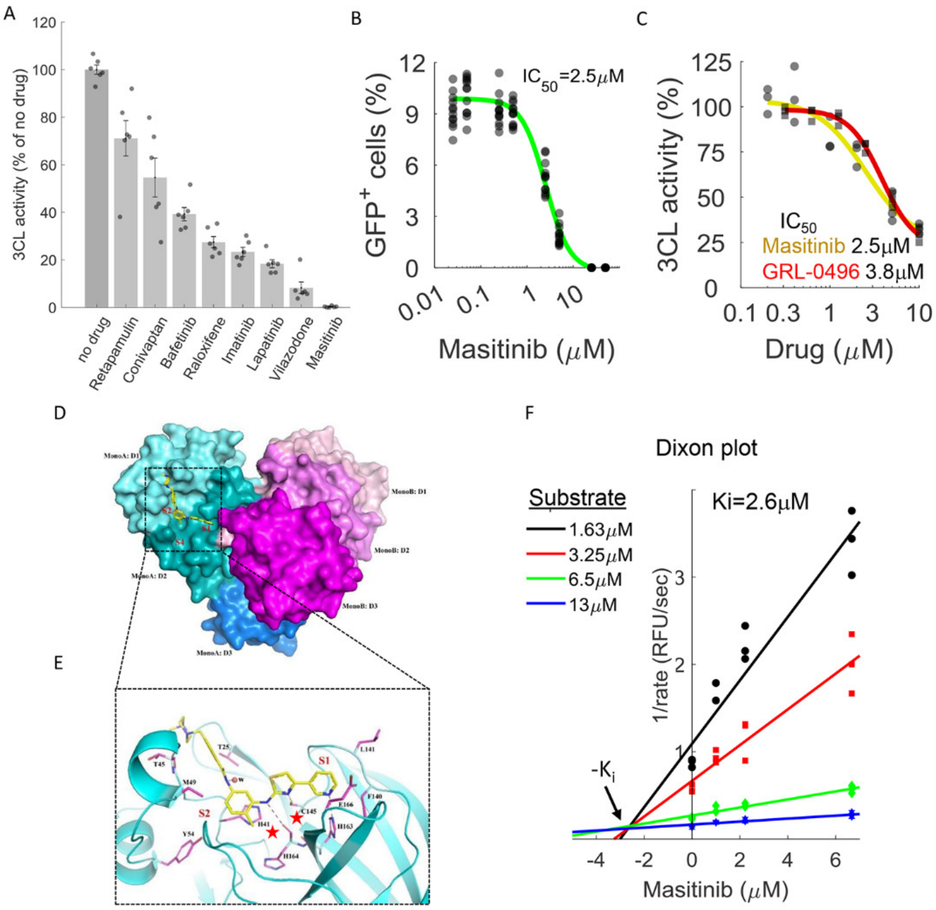

Fig. 3. Masitinib inhibits SARS-CoV-2 3CLpro enzymatic activity. (**A**) A FlipGFP reporter assay was performed to screen for potential inhibition of 3CL by the identified drugs at a single concentration (10μM). Shown are the drugs that showed a statistically significant reduction in 3CLpro activity (p-value<0.05, one-tailed *t* test, FDR-corrected). n=6. The data for the remaining tested drugs is shown in fig. S4. Individual measurements are shown in circles. Bars depict mean ± s.e. (**B**) Dose-response curve for 3CL inhibition by masitinib using the FlipGFP reporter assay, n = 6. Individual measurement shown as circles. (**C**) Dose-response curve for 3CL inhibition by masitinib (yellow) and GRL-0496 (red) using a luciferase reporter assay, n = 3. Induvial measurement shown as circles (masitinib) or squares (GRL-0496) (**D**) The dimer formation, domain structure, and masitinib binding site of SARS-CoV-2 3CL. Domains I, II and III (D1-D3) of the monomer A of a 3CL dimer are colored in cyan, teal and light blue, respectively. The corresponding three domains of monomer B are colored in light pink, magenta and purple. In monomer A, masitinib is drawn in stick format, bound to the active site. The location of the three binding pockets S1, S2, and S4 are marked in red. (**E**) A ribbon diagram showing details of some interactions formed between masitinib and 3CL at the active site. Masitinib is drawn in stick format with its C atoms colored in yellow. Key pocket forming or interacting residues of 3CL are also presented in stick format with their C atoms colored in purple. Hydrogen bonds are drawn in black dashed lines. The sites of binding pockets S1 and S2 are marked in red. The two catalytic residues are marked by red stars. (**F**) Dixon plot, showing the rate of 3CL activity in the presence of different substrate and masitinib concentrations. n=3.

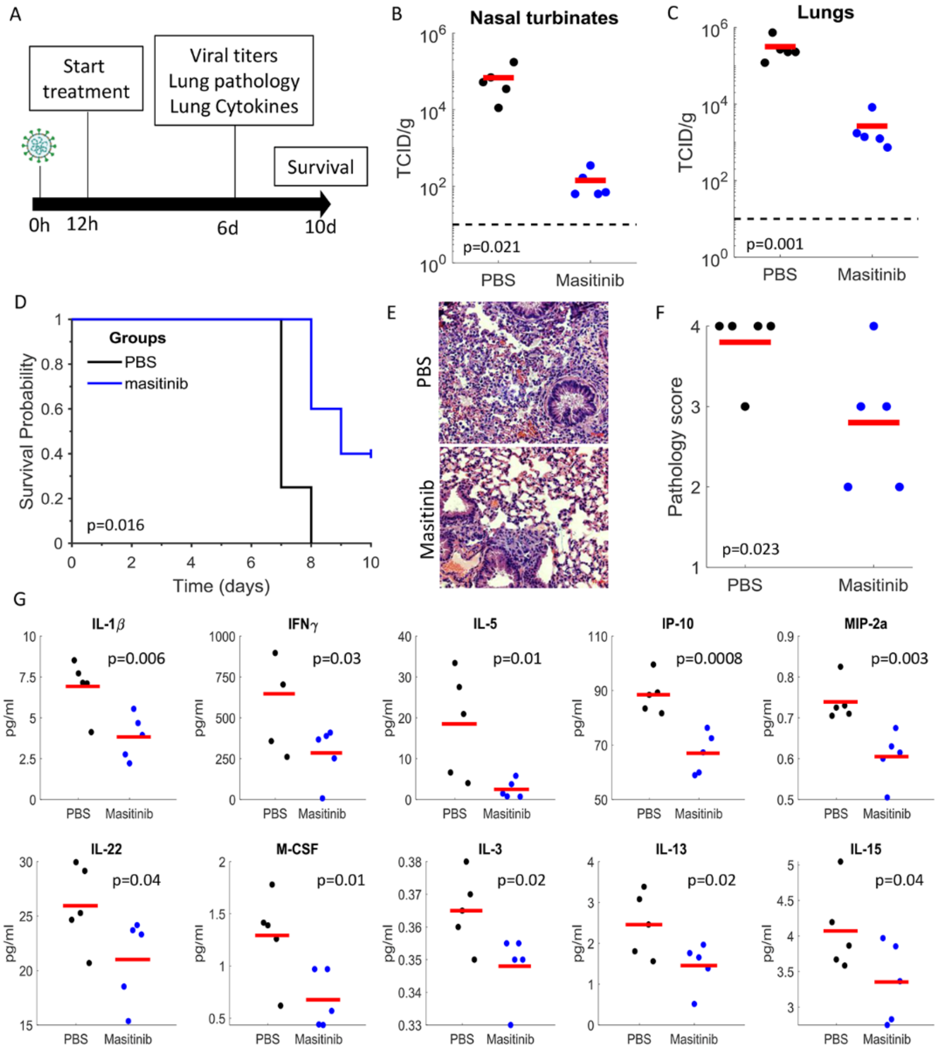

Fig. 4. Masitinib inhibits SARS-CoV-2 replication in mice. (**A**) Schematic diagram of experiment. (**B** and **C**) SARS-CoV-2 infectious virus measurements in the nose (B) and lungs (C) of PBS (black) or masitinib (blue) treated mice at day 6 post infection. n=5 mice per group. Red lines are the mean values. P-values shown in figure (one-tailed *t* test). (**D**) Kaplan-Meir curves assessing mice survival after PBS (black, n=4) or masitinib (blue, n=5) treatment post-infection. P-value shown in figure (log-rank test). (**E**) Representative images of lung histology (H&E) stain at 6 days post infection. (**F**) Lung pathology score on day 6 post infection. Tissues were blindly scored on a scale of 0-4 by an expert veterinarian pathologist. n=5 mice per group. P-value shown in figure (one-tailed *t* test). (**G**) Cytokine levels in the lung of infected mice treated with PBS (black) or masitinib (blue) at day 6 post infection. n=5 mice per group. Red lines are the mean values. P-values shown in figure (one-tailed *t* test). Experiment was performed once.
